# Supplementary material for: Text-based predictions of COVID-19 diagnosis from self-reported chemosensory descriptions
Source: Commun Med (Lond). 2023 Jul 27;3:104. doi: 10.1038/s43856-023-00334-5 (PMC10374642; doi:10.1038/s43856-023-00334-5)
Supplement: Supplementary file 2 — Supplementary Information [file 43856_2023_334_MOESM2_ESM.pdf]

Supplementary Information

Li *et al*

Text-based predictions of COVID-19 diagnosis from self-reported chemosensory descriptions.

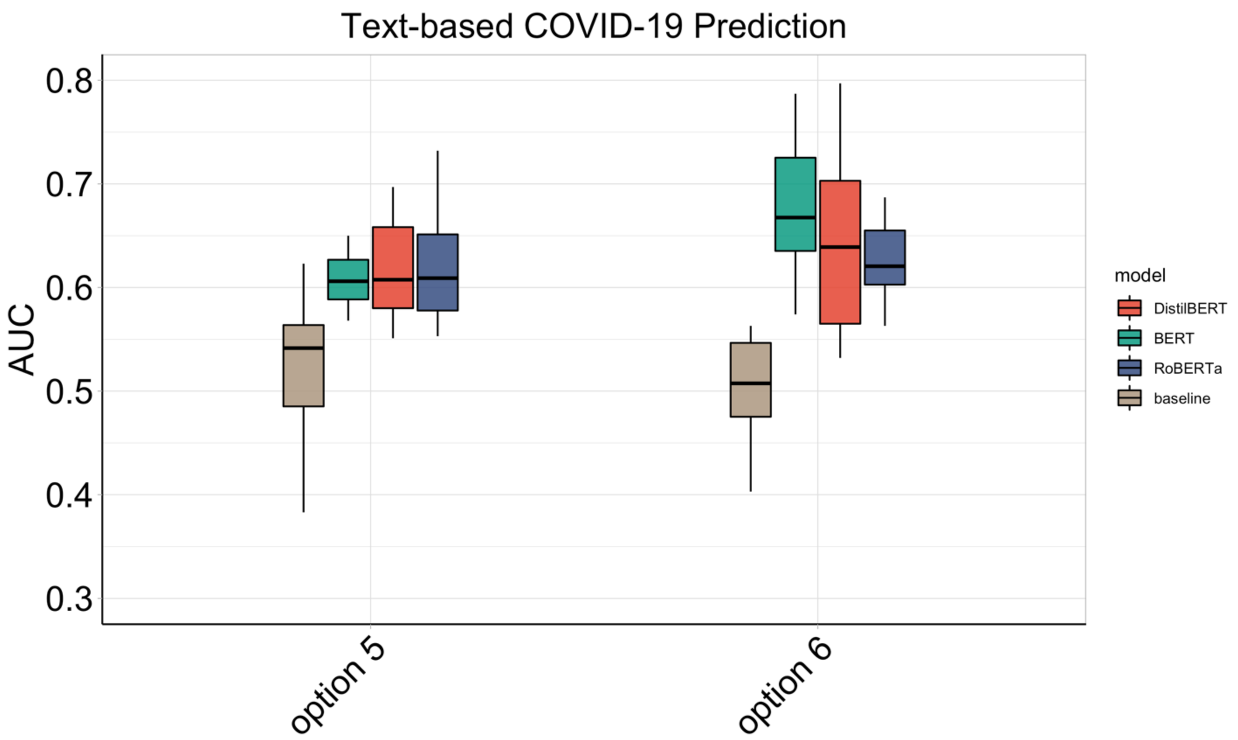

Supplemental Figure 1. The predictive performance comparison of DistilBERT, BERT, RoBERTa and baseline.

The AUC-ROCs of 10-fold cross-validations experiments are shown as boxplots for option 5 class and option 6 class predictions. In each boxplot, the horizontal line represents the median value. The whiskers represent the maximum and minimum values, whereas the bottom and top of boxes represent the first (25%) and third (75%) quartile.

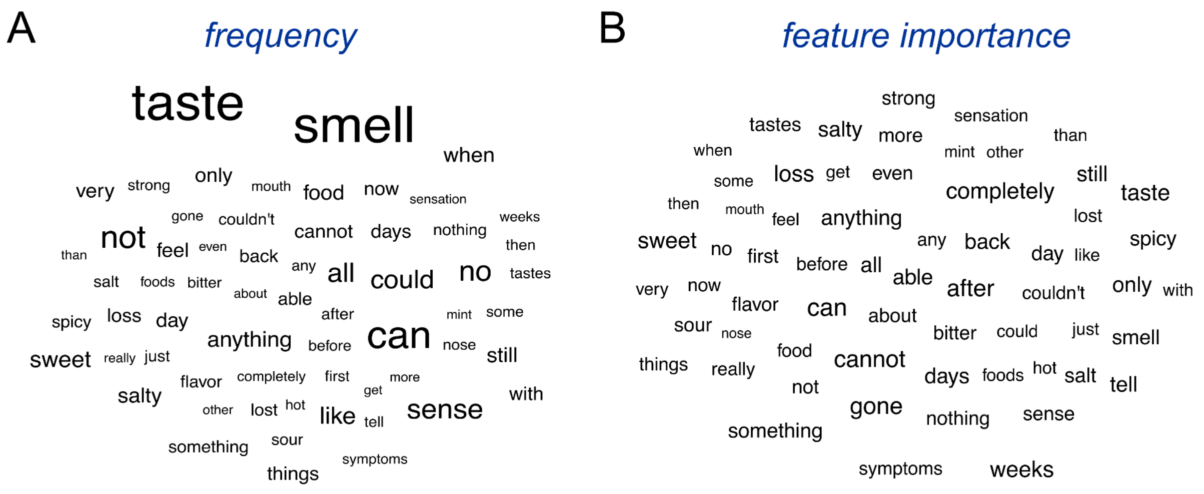

Supplemental Figure 2. Feature importance analysis of key words in predicting COVID-19 **A.** The frequency of highly occurring words is shown as a word cloud for the option 5 (with respiratory symptoms) class model. The occurrence frequency is scaled to the size of the word. **B.** The contributions of highly occurring words in predicting COVID-19 is shown as a word cloud for the option 5 class model. The feature importance, or absolute SHAP value, is scaled to the size of the word.

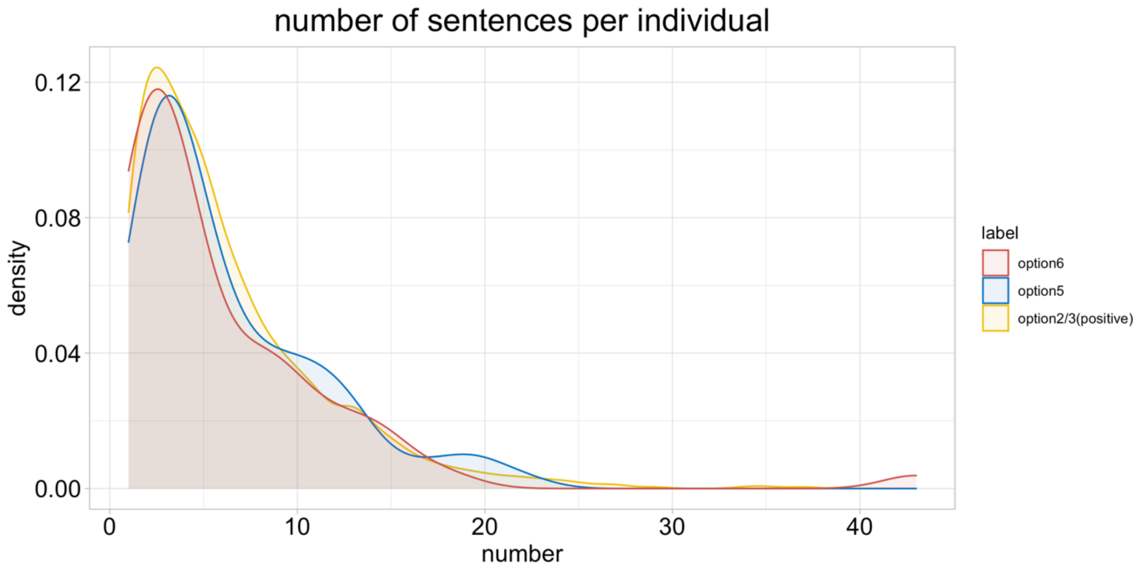
number of sentences per individual
0.12
0.08
0.04
0.00
density
0
10
20
30
40
number
label
option6
option5
option2/3(positive)

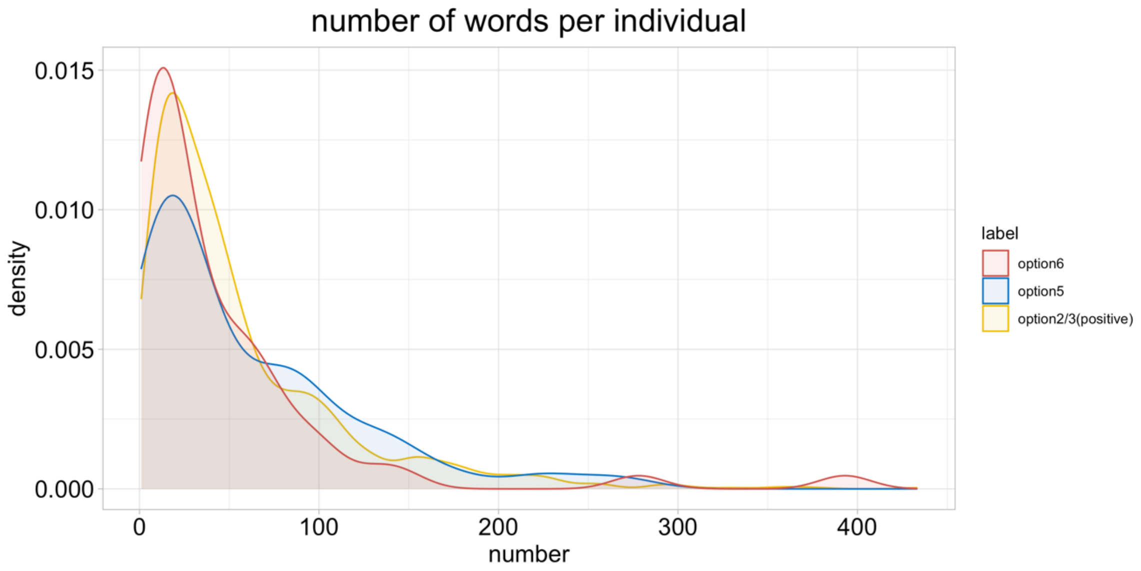
number of words per individual
0.015
0.010
0.005
0.000
density
0
100
200
300
400
number
label
option6
option5
option2/3(positive)

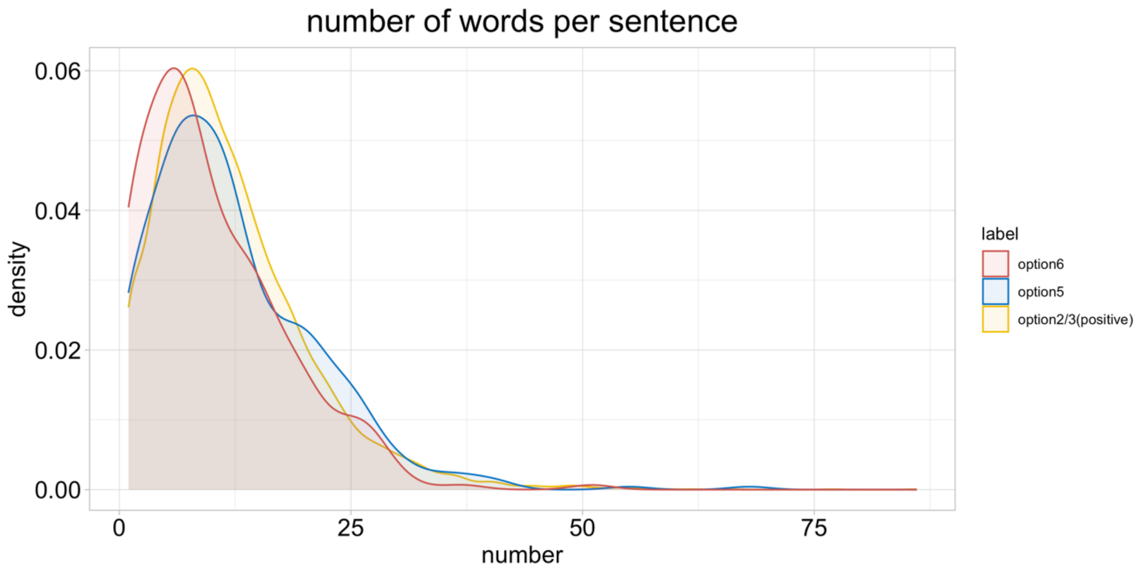
number of words per sentence
0.06
0.04
0.02
0.00
density
0
25
50
75
number
label
option6
option5
option2/3(positive)

Supplemental Figure 3. The density distributions of the number of sentences and words in the text description of perception changes.

We analyzed three categories of participants: (1) Option 6: COVID-19 negative without symptoms, (2) Option 5: COVID-19 negative with symptoms, and (3) Option 2/3: COVID-19 positive. For each category, we calculated A. the number of sentences per individual, B. the number of words per individual, and C. the number of words per sentence.

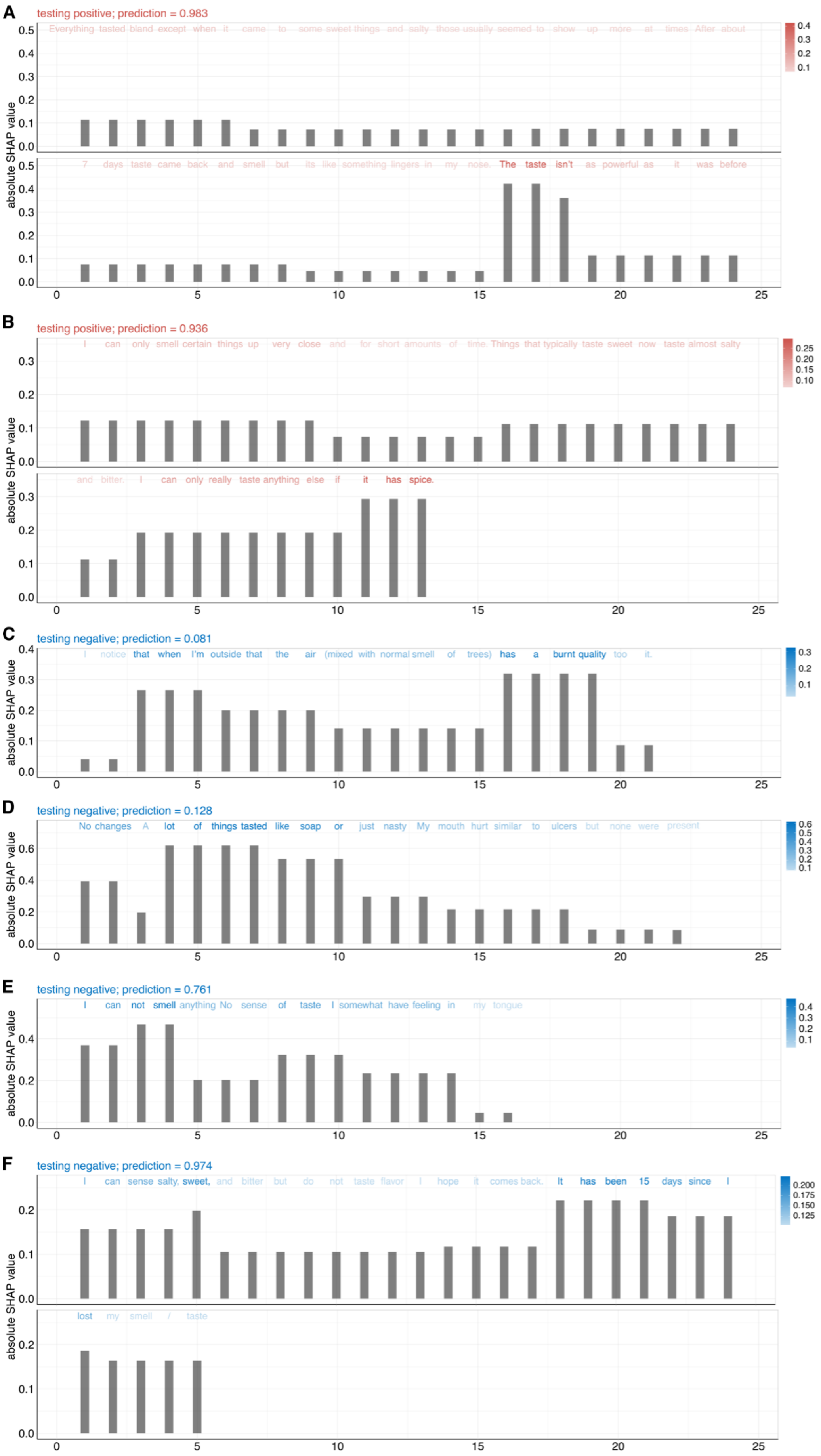

A
testing positive; prediction = 0.983
Everything tasted bland except when it came to some sweet things and salty those usually seemed to show up more at times After about
7 days taste came back and smell but its like something lingers in my nose. The taste isn't as powerful as it was before
absolute SHAP value
B
testing positive; prediction = 0.936
I can only smell certain things up very close and for short amounts of time. Things that typically taste sweet now taste almost salty
and bitter. I can only really taste anything else if it has spice.
absolute SHAP value
C
testing negative; prediction = 0.081
I notice that when I'm outside that the air (mixed with normal smell of trees) has a burnt quality too it.
absolute SHAP value
D
testing negative; prediction = 0.128
No changes A lot of things tasted like soap or just nasty My mouth hurt similar to ulcers but none were present
absolute SHAP value
E
testing negative; prediction = 0.761
I can not smell anything No sense of taste I somewhat have feeling in my tongue
absolute SHAP value
F
testing negative; prediction = 0.974
I can sense salty, sweet, and bitter but do not taste flavor I hope it comes back. It has been 15 days since I
lost my smell / taste
absolute SHAP value

Supplemental Figure 4. Feature importance analysis of input text responses in predicting COVID-19 positive and negative examples.

The height of the bar plot under each word as well as the color transparency correspond to the absolute SHAP value in predicting COVID-19. The SHAP values were calculated from the option 5 class model on the testing dataset. **A-B.** The text responses of two COVID-19 negative examples are shown in red. **C-F.** The text responses of four COVID-19 negative examples are shown in blue.

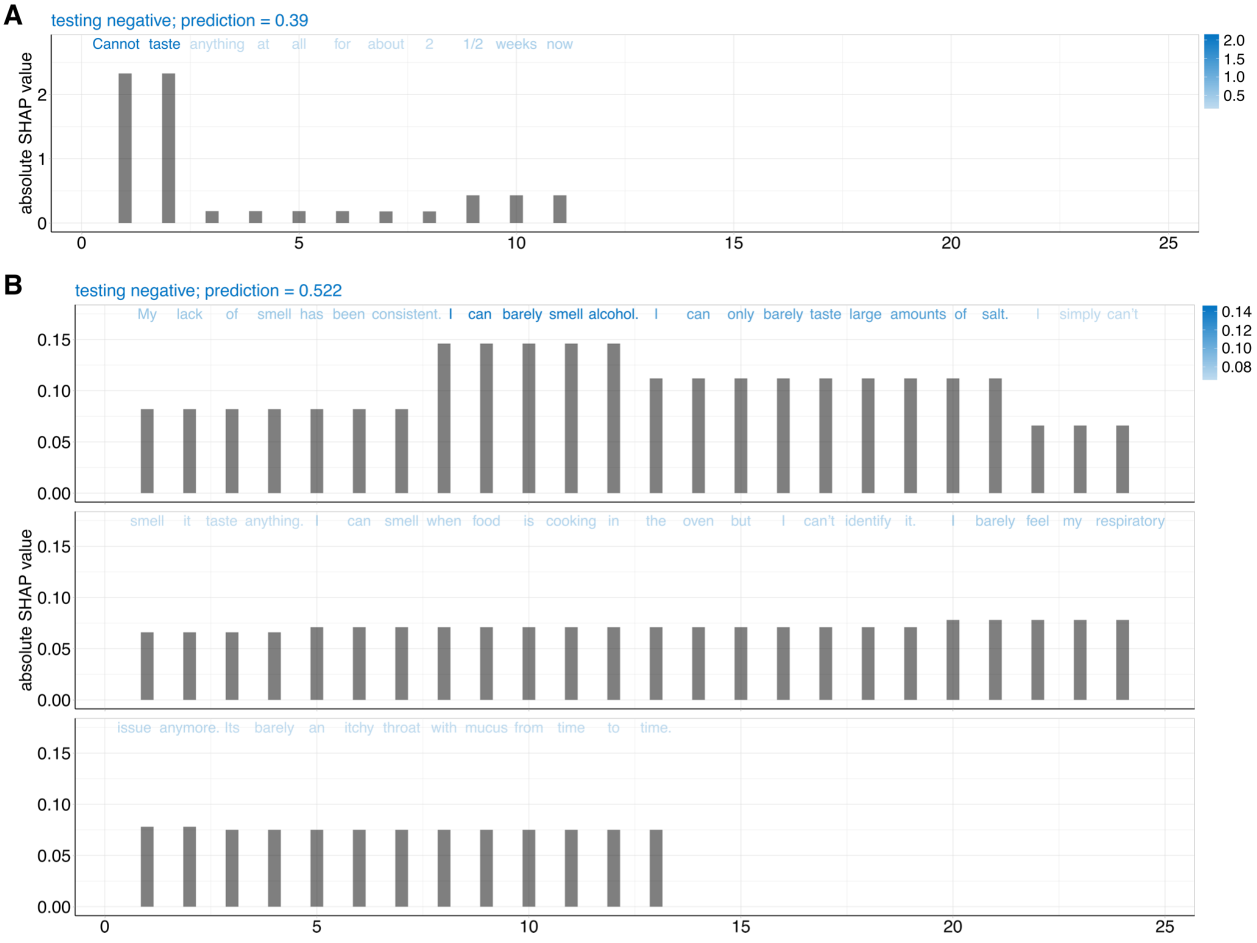

Supplemental Figure 5. Feature importance analysis of input text responses in predicting COVID-19 positive and negative examples.
The height of the bar plot under each word as well as the color transparency correspond to the absolute SHAP value in predicting COVID-19. The SHAP values were calculated from the option 6 class model on the testing dataset. **A-B.** The text responses of two COVID-19 negative examples are shown in blue.
