# Supplementary material for: Text-based predictions of COVID-19 diagnosis from self-reported chemosensory descriptions
Source: Commun Med (Lond). 2023 Jul 27;3:104. doi: 10.1038/s43856-023-00334-5 (PMC10374642; doi:10.1038/s43856-023-00334-5)
Supplement: Supplementary file 3 — Description of Additional Supplementary Files [file 43856_2023_334_MOESM3_ESM.docx]

**Data for Figure 2.** AUC values resulting from the fine tunning of the indicated models following a 10 fold cross-validation scheme for option 5 class model and option 6 class model (shown in Figure 2 and Supplementary Figure 1).

**SHAP values for used words.** The word counts and absolute SHAP values of highly occurring words in the option 5 class model and option 6 class model.

**Statistics of word/sentence use.** The statistics of sentences and words used in the text response.

**Data for Figure 4.** SHAP values for each of the words in 6 examples resulting from fine-tuning a DistillBERT model for option 5 class model (shown in Supplementray Figure 4) and option 6 class model (shown in Figure 4).
